# Kynurenine Is the Main Metabolite of Tryptophan Degradation by Tryptophan 2,3-Dioxygenase in HepG2 Tumor Cells

**DOI:** 10.3390/jcm11164794

**Published:** 2022-08-16

**Authors:** Hani Oweira, Imad Lahdou, Stefan Mehrle, Elias Khajeh, Rajan Nikbakhsh, Omid Ghamarnejad, Peter Terness, Christoph Reißfelder, Mahmoud Sadeghi, Ali Ramouz

**Affiliations:** 1 Department of Surgery, Medical Faculty Mannheim, University of Heidelberg, 68167 Mannheim, Germany; 2 Department of Transplantation Immunology, University of Heidelberg, 69120 Heidelberg, Germany; 3 Department of General, Visceral, and Transplantation Surgery, University of Heidelberg, 69120 Heidelberg, Germany

**Keywords:** liver cell, tryptophan, kynurenine

## Abstract

There are two main enzymes that convert tryptophan (Trp) to kynurenine (Kyn): tryptophan-2,3-dioxygenase (TDO) and indoleamine 2,3-dioxygenase (IDO). Kyn accumulation can promote immunosuppression in certain cancers. In this study, we investigated Trp degradation to Kyn by IDO and TDO in primary human hepatocytes (PHH) and tumoral HepG2 cells. To quantify Trp-degradation and Kyn-accumulation, using reversed-phase high-pressure liquid chromatography, the levels of Trp and Kyn were determined in the culture media of PHH and HepG2 cells. The role of IDO in Trp metabolism was investigated by activating IDO with IFN-γ and inhibiting IDO with 1-methyl-tryptophan (1-DL-MT). The role of TDO was investigated using one of two TDO inhibitors: 680C91 or LM10. Real-time PCR was used to measure TDO and IDO expression. Trp was degraded in both PHH and HepG2 cells, but degradation was higher in PHH cells. However, Kyn accumulation was higher in the supernatants of HepG2 cells. Stimulating IDO with IFN-γ did not significantly affect Trp degradation and Kyn accumulation, even though it strongly upregulated IDO expression. Inhibiting IDO with 1-DL-MT also had no effect on Trp degradation. In contrast, inhibiting TDO with 680C91 or LM10 significantly reduced Trp degradation. The expression of TDO but not of IDO correlated positively with Kyn accumulation in the HepG2 cell culture media. Furthermore, TDO degraded L-Trp but not D-Trp in HepG2 cells. Kyn is the main metabolite of Trp degradation by TDO in HepG2 cells. The accumulation of Kyn in HepG2 cells could be a key mechanism for tumor immune resistance. Two TDO inhibitors, 680C91 and LM10, could be useful in immunotherapy for liver cancers.

## 1. Introduction

One of the key pathways implicated in chronic liver diseases and liver malignancies is tryptophan (Trp) degradation, which leads to kynurenine (Kyn) accumulation [1,2,3]. Under normal physiological conditions, Trp is degraded by two main enzymes—indoleamine 2,3-dioxygenase (IDO) and tryptophan-2,3-dioxygenase (TDO)—to produce a variety of metabolites, including kynurenine (Kyn) [4]. The degradation of Trp into Kyn has the potential to stimulate tumor growth, as well as influence the immune system by limiting T lymphocyte proliferation and promoting T-cell differentiation [5,6,7].

In recent years, researchers have become more interested in the role of the kynurenine pathway (KP) in liver diseases. High Kynurenine-Pathway (KP) activity has been measured in chronic liver disorders such as primary biliary cirrhosis, HCV-associated chronic hepatitis, and liver cirrhosis [1,2]. According to Claria et al., KP activity could be higher in individuals with acute decompensation and acute-on-chronic liver failure, and has been linked to the outcomes of cirrhotic patients [3,7]. Additionally, Kynurenine accumulation promotes immunosuppression in liver cancer [7], and it has been shown that IDO and TDO are overexpressed in hepatocellular carcinoma (HCC) tissue. This overexpression promotes tumoregenesis by increasing tumor size, vascular invasion, and tumor differentiation [8,9], and by inhibiting antitumor immunoreactivity, which reduces survival in HCC patients [10]. Therefore, inhibiting IDO and TDO activity to reduce Trp degradation and Kyn accumulation may represent an effective approach for HCC immunotherapy [7]. However, the exact mechanisms of this potential therapeutic pathway in HCC cells remain unclear. Hepatic cell lines provide the best available environment to evaluate the exact correlation between Kyneurine and its metabolites in HCC patients [11]. However, due to their high cost, invasiveness, and the diminished activity of numerous important enzymes, immortalized hepatic cell lines are frequently utilized instead of liver biopsies in much research. Furthermore, there is no method for keeping liver biopsies in culture during time-consuming research [12]. Predictions of drug metabolism and toxicity are studied in in vitro liver models, including primary human hepatocytes (PHH) [13]. Due to the proper transportation and signaling pathways of the PHH cells, and their total maintenance of metabolism, they have commonly been used to study liver function and drug toxicity. On the other hand, HepG2 cells were the first hepatic cell lines to show the main properties of hepatocytes, hepatoma, and hepatocellularcarcinoma (HCC) [14]. To compare the Kyn pathway in normal liver cells and tumor cells, we studied the involvement of TDO and IDO in Trp degradation and Kyn accumulation in primary human hepatocytes (PHH) and the tumoral cell line HepG2.

## 2. Materials and Methods

### 2.1. Cell Culture

HepG2 cells [15,16] and PHH cells [17] (Nunc, Wiesbaden, Germany) were cultured as previously described. PHH and HepG2 cells were cultured in William’s medium with increasing concentrations of Trp (60, 120, and 240 µmol/L). Trp and Kyn (Roche Diagnostics, Mannheim, Germany) levels in cultivated PHH and HepG2 cell supernatants were examined at 24 h, 48 h, and 72 h. In the next step, we also investigated the maximum Trp degradation in HepG2 cells by culturing these cells for 72 h in William’s medium containing higher amounts of Trp (480 µmol/L, 960 µmol/L, and 1920 µmol/L). To determine whether IDO is responsible for Trp breakdown, we activated IDO with IFN-γ (Sigma Aldrich) and inhibited IDO with 1-methyl-tryptophan (1-DL-MT) (Nunc, Wiesbaden, Germany) [18,19] in PHH and HepG2 cells. In these experiments, the cells were cultured for 72 h under one of the following conditions: no treatment, stimulated with IFN-γ, treated with IFN-γ + 1-DL-MT, and treated with 1-DL-MT (R&D System, Wiesbaden-Nordenstadt, Germany) alone. After 72 h, the amounts of Trp and Kyn in the supernatants were determined. To determine whether TDO is responsible for Trp breakdown, we cultured cells with the TDO inhibitors LM10 and 680C91 (R&D System, Wiesbaden-Nordenstadt, Germany). In these experiments, HepG2 cells were treated with increasing amounts of 680C91 (10, 20, 40, and 80 µmol/L) and LM10 (25, 50, and 75 µmol/L) in a medium containing 240 µM Trp for 72 h before measuring Trp and Kyn levels in the supernatant. To investigate which Trp isoform (L-Trp or D-Trp) is degraded by TDO, we cultured HepG2 cells in media containing 60, 120, or 240 µM of L-Trp or D-Trp for 72 h, and measured the Trp and Kyn levels after 72 h.

### 2.2. Reversed Phase High Pressure Liquid Chromatography (RP-HPLC)

The concentrations of Trp and Kyn were quantified in culture supernatants using RP-HPLC as described previously [20]. To eliminate the remaining proteins, the sample was treated with 10% (*v*/*v*) 2.4 M perchloric acid and incubated for 5 min at room temperature. After centrifugation at 5850 rpm for 15 min at 4 °C, the supernatants were placed in fresh vials. Then, 100 µL of filtered supernatant was loaded into a C-18 column after the pH was adjusted to 7.0. (Supelco, Aschaffenburg, Germany). PBS was used to elute the samples for 30 min. Trp’s natural fluorescence was measured at 285 nm excitation and 360 nm emission, whereas Kyn was measured at 230 nm using UV absorption. The retention durations of standard compounds were used to calculate the peaks for Kyn and Trp. The ratios of the compound’s peak areas to those of the internal standard were used to quantify it [20]. To differentiate between two Trp isoforms, L-Trp (L-Trp) and D-Trp (D-Trp), the peaks of L-Trp and D-Trp were identified by comparison with the retention time of previously determined standard compounds, and quantification was based on the ratios of the compound’s peak areas to the internal standard, with a L-Trp retention time equal to 1.197 min and a D-Trp retention time equal to 1.564 min.

### 2.3. Real-Time Polymerase Chain Reaction

Total RNA was extracted and measured in a NanoDrop spectrophotometer using RNeasy-plus Mini Kit spin columns (Qiagen Gmbh, Hilden, Germany) (ND-1000; NanoDrop Technologies, Wilmington, DE, USA). Reverse transcription of one microgram of total RNA to cDNA was performed, and first strand synthesis was performed with SuperScript III and Super Mix for quantitative reverse transcription PCR (Invitrogen, Darmstadt, Germany), according to the manufacturer’s protocol. Using Applied Biosystems-7500 real-time PCR equipment, real-time PCR was performed using the SYBR GreenER Two-Step qRT-PCR kit Universal (Invitrogen, Darmstadt, Germany) (Applied Bioscience, Darmstadt, Germany). The endogenous control gene glycerinaldehyde-3-phosphate-dehydrogenase (GAPDH) was utilized to adjust for variable RNA starting quantities.

The following oligonucleotide sequences were used in the PCR reactions: GAPDH: sense primer 5′-GAA GGT GAA GGT CGG AGT-3′, antisense primer 5′-AGA TGG TGA TGG GAT TTC-3′; IDO: sense primer 5′-CAG CTG CTT CTG CAA TCA AA-3′, antisense primer 5′-AGC GCC TTT AGC AAA GTG TC-3′; TDO: sense primer 5′-CAA ATC CTC TGG GAG TTG GA-3′, antisense primer 5′-GTG CAT CCG AGA AAC AAC CT-3′. Initial denaturation at 95 °C for 10 min was followed by 40 cycles of two-step PCR (denaturation at 95 °C for 10 s, annealing and extension at 60 °C for 60 s). Products were assessed by melting curve analysis, and relative quantification was performed as previously described [21].

### 2.4. Statistical Analysis

GraphPad Prism 7 software was used to examine group comparisons using the *t*-test and one-way and two-way ANOVA, followed by Tukey’s post hoc test (San Diego, CA, USA). Statistical significance was defined as a *p* value of less than 0.05.

## 3. Results

### 3.1. Trp Degradation to Kyn in PHH and HepG2 Cells

**Figure 1:**
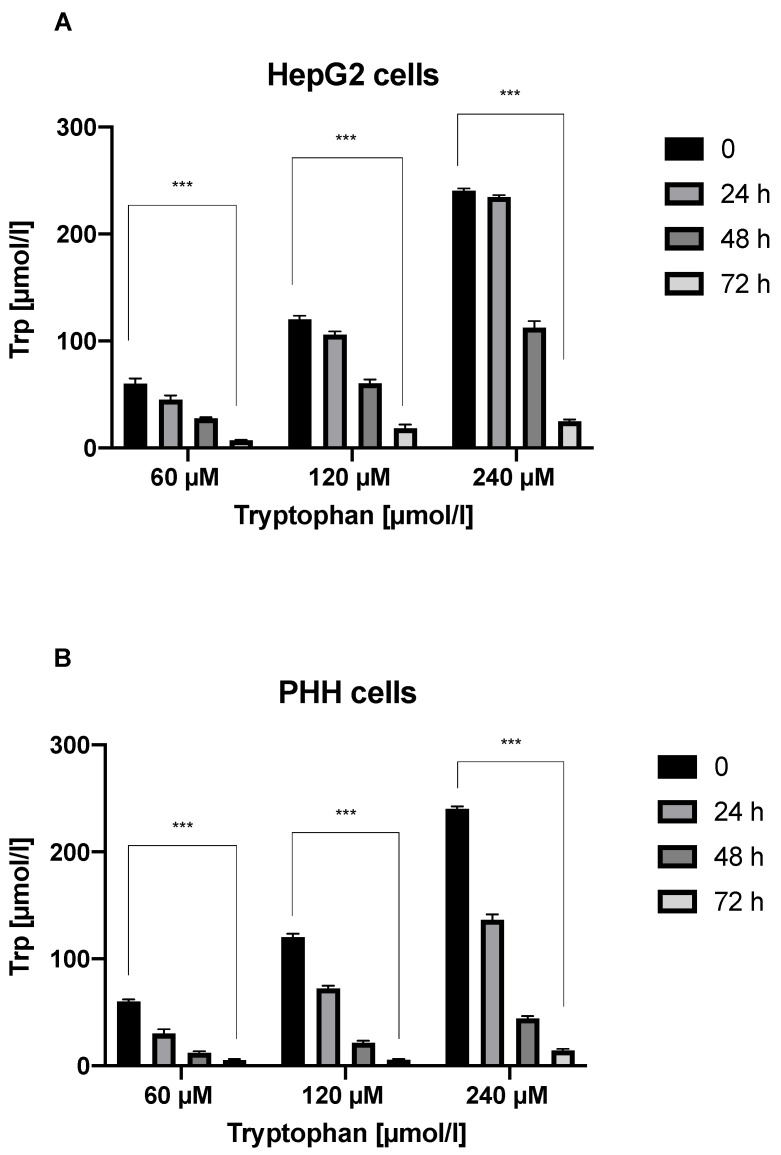
Trp levels in HepG2 (**A**) and PHH (**B**) cell supernatants over time. *** *p* < 0.001.

**Figure 2:**
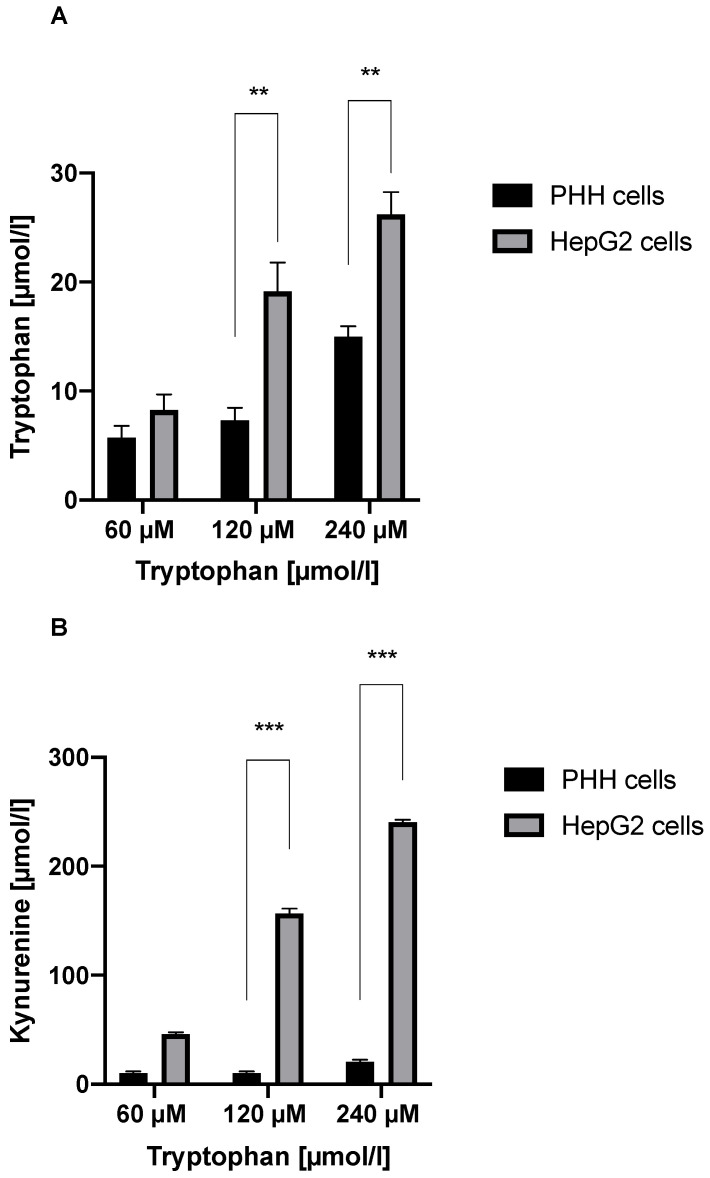
Trp degradation and Kyn accumulation in different hepatocytes at 72 h. (**A**) The comparison of Trp degradation between PHH cells and HepG2 cells. (**B**) The comparison of Kyn accumulation between PHH cells and HepG2 cells. ** *p* < 0.01, and *** *p* < 0.001.

Trp was degraded in PHH and HepG2 cells, as shown by lower Trp levels at 72 h than at 24 h and 48 h (Figure 1A,B). Trp degradation was significantly higher in PHH cells than in HepG2 cells at 72 h (*p* < 0.05) (Figure 2A). Kyn levels were substantially greater in HepG2 cell supernatants than in PHH cell supernatants at 72 h. (*p* < 0.01) (Figure 2B).

**Figure 3:**
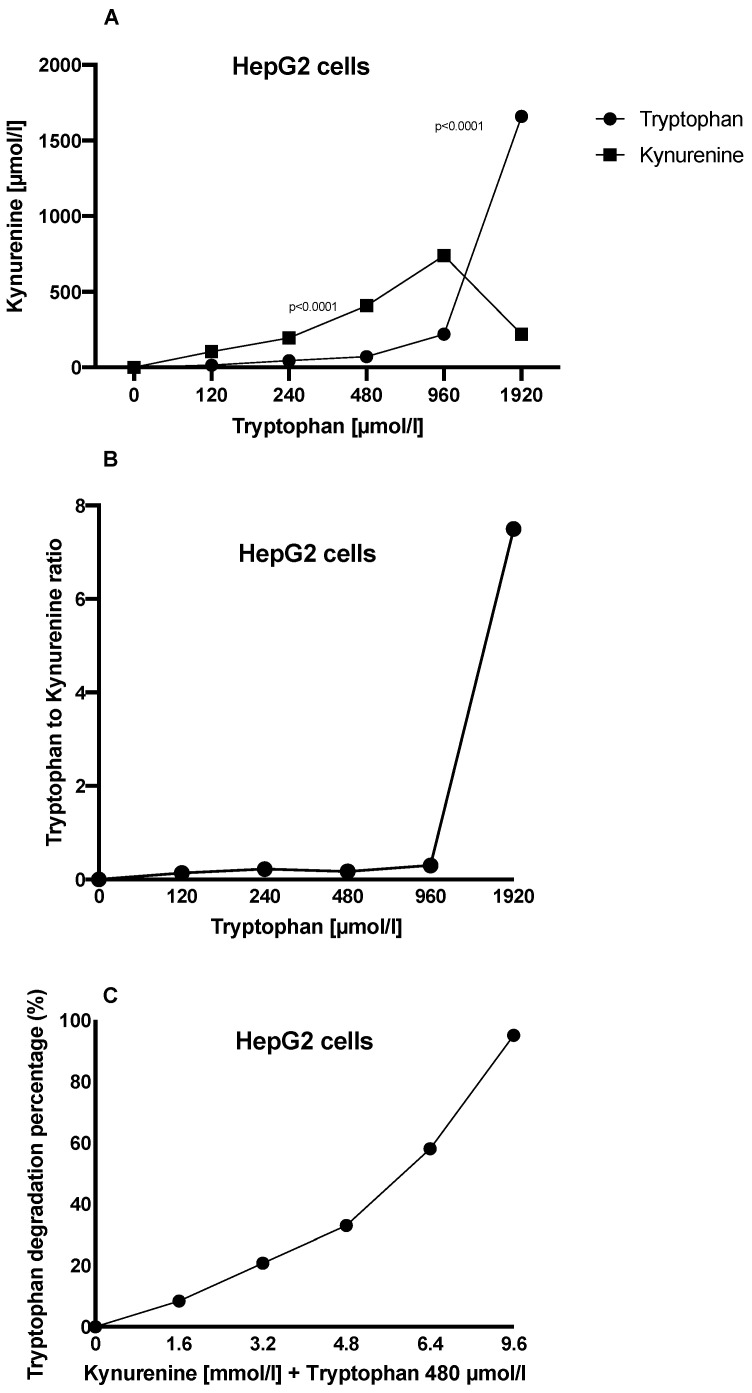
(**A**) Kyn accumulation and Trp degradation for different concentration levels of Trp in HepG2 cells. (**B**) The Trp to Kyn ratio increases as higher levels of kynurenine suppress Trp degradation. (**C**) The percentage of Trp degradation in HepG2 cells cultured in 480 µM Trp and various concentrations of Kyn for 72 h.

We also investigated the maximum Trp degradation in HepG2 cells. Trp degradation and Kyn generation were lower in HepG2 cells cultured in media containing 1920 µM Trp than in cells cultured in media containing 480 µM and 960 µM Trp. We believe that high Trp levels in the medium inhibited cell metabolism, but further experiments are needed to confirm this. HepG2 cells cultured for 72 h in a medium containing 960 µM Trp degraded around 700 µM Trp and accumulated the same amount of Kyn (Figure 3A). We believe that Kyn accumulation is a way for tumor cells to evade the host immune system’s reaction. (Figure 3A). Additionally, our analysis suggests that the Trp to Kyn ratio increases as higher levels of kynurenine suppress Trp degradation (Figure 3B).

### 3.2. Extremely High Levels of Kyn Affect Trp Degradation in Hepatic Cells

To investigate this further, we cultured HepG2 cells in a medium with 480 µmol/L Trp and various concentrations of Kyn (1.0, 2.5, 5.0, 7.5, and 10.0 mmol/L) for 72 h. Trp degradation in HepG2 cells was reduced by 3% in the presence of 1.0 mmol/L Kyn and by 6.7% in the presence of 2.5 mmol/L Kyn. Trp degradation was reduced even further in Kyn concentrations of 5.0 mmol/L and above (by 34.4% in 5.0 mmol/L, by 63.25% in 7.5 mmol/L, and by >99% in 10.0 mmol/L Kyn) (Figure 3C).

### 3.3. TDO but Not IDO Degrades Trp in PHH and HepG2 Cells

**Figure 4:**
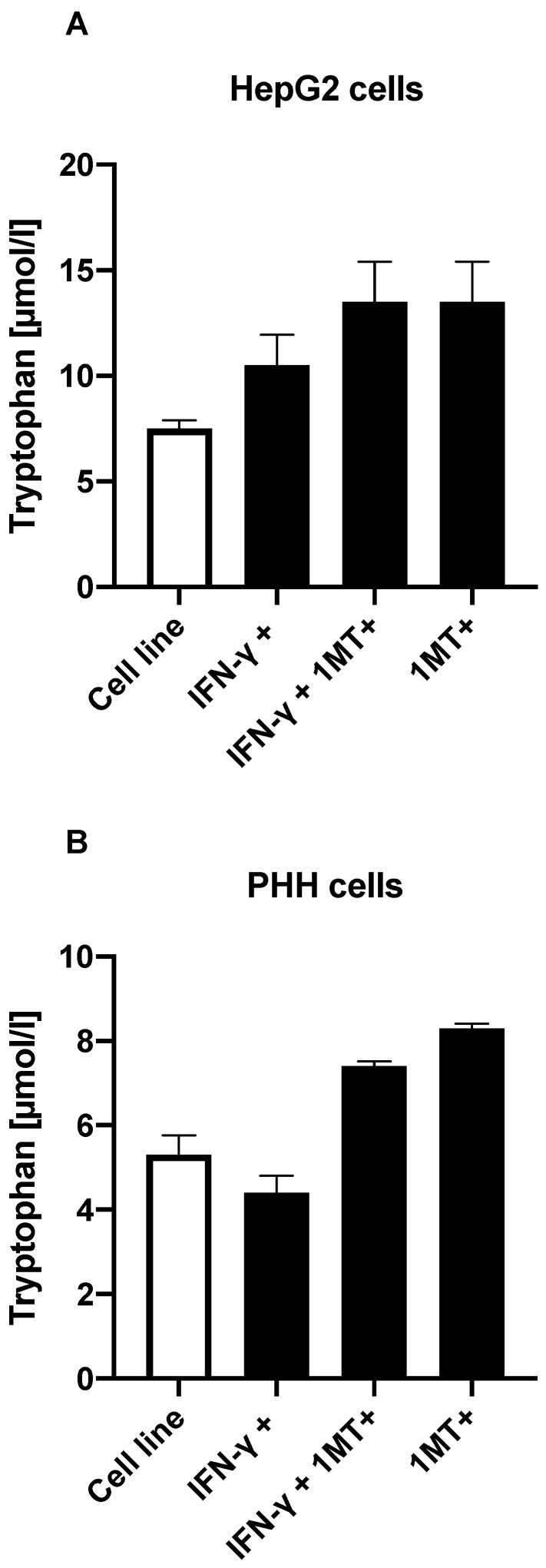
(**A**) Trp degradation in HepG2 cells treated with IFN-γ to activate IDO and/or with 1-DL-MT to inhibit IDO. (**B**) Trp degradation in PHH cells treated with IFN-γ to activate IDO and/or with 1-DL-MT to inhibit IDO.

We investigated whether IDO is responsible for Trp breakdown in a culture medium containing 60 µmol Trp by treating cells with IFN-γ to activate IDO, and with 1-DL-MT to inhibit IDO (21, 22); the amounts of Trp and Kyn in the supernatant were then measured. Stimulation of IDO with IFN-γ did not significantly alter Trp degradation (Figure 4A,B), even though IFN-γ increased IDO expression by more than 1400 times in the HepG2 cells (see Section 3 and Section 4). These results show that Trp is not degraded by IDO in these cells.

**Figure 5:**
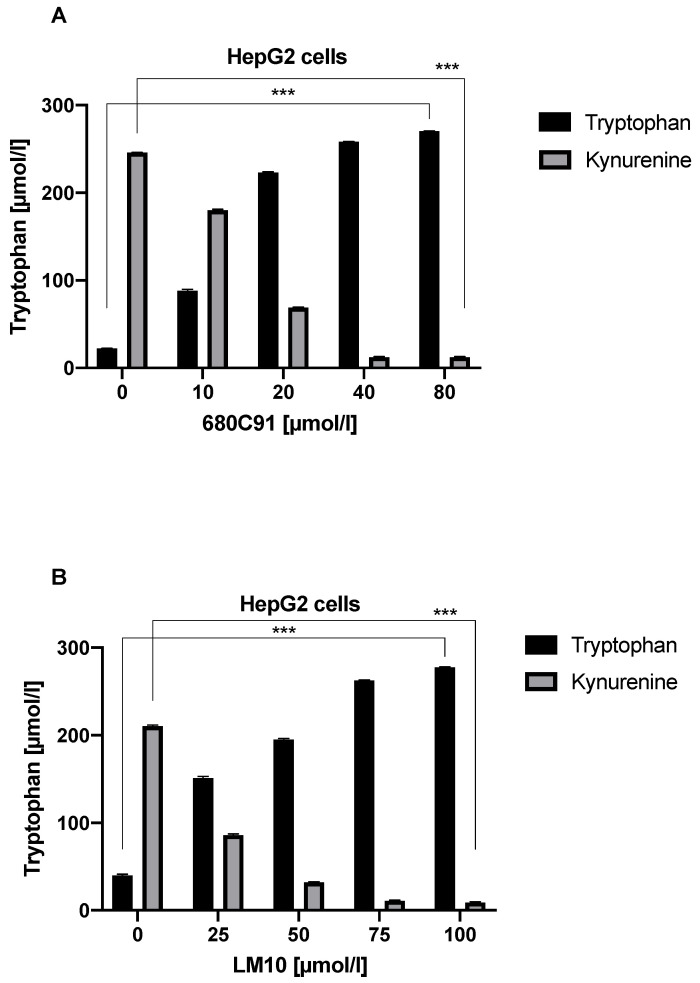
The effect of TDO inhibition with 680C91 (**A**) and LM10 (**B**) on Trp degradation in HepG2 cells. *** *p* < 0.001.

To determine whether TDO is responsible for Trp breakdown, we cultured cells in the presence of the TDO inhibitors LM10 and 680C91. Kyn was not present in the supernatant of cells cultured in 40 µmol/L 680C91 or 75 µmol/L LM10. Moreover, there was a direct correlation between the concentration of 680C91/LM10 and levels of Kyn, suggesting that TDO is involved in the degradation of Trp to Kyn (Figure 5A,B).

### 3.4. Expression of IDO and TDO in Tumoral Hepatic Cells

**Figure 6:**
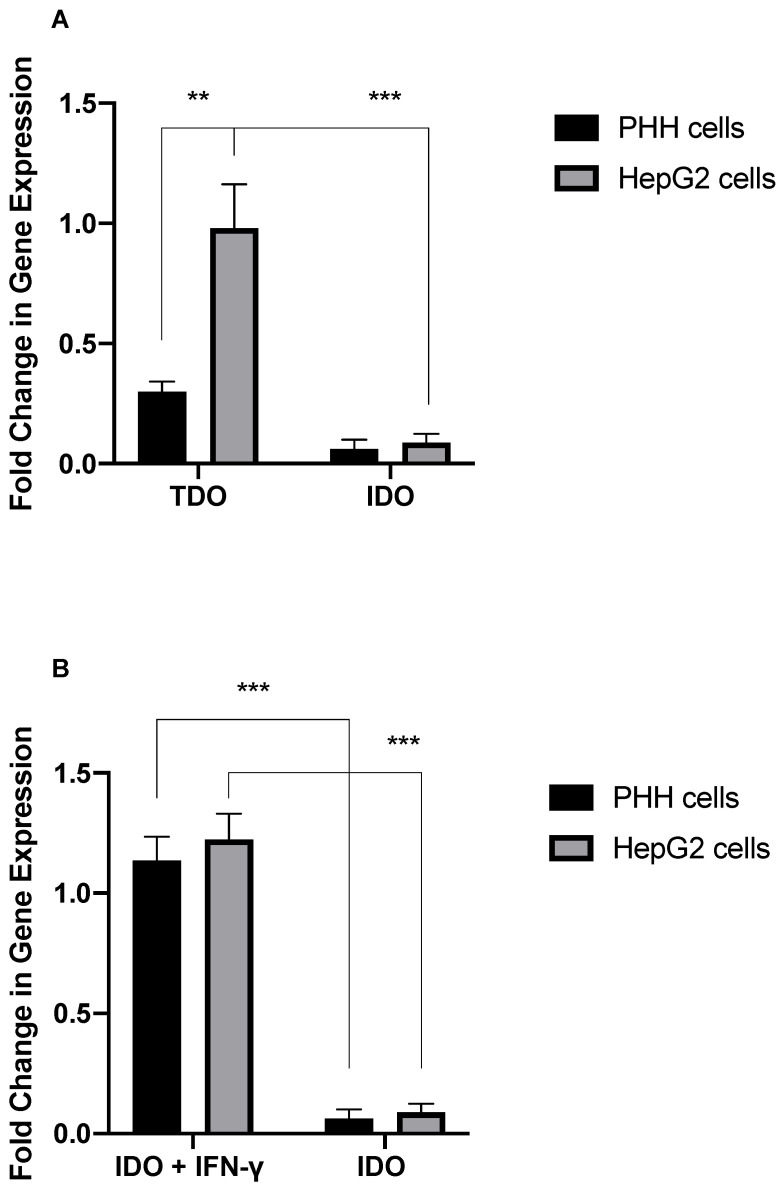
(**A**) TDO and IDO expression in PHH and HepG2 cells. (**B**) IDO expression in PHH and HepG2 cells treated with IFN-γ. ** *p* < 0.01, and *** *p* < 0.001.

Using real-time PCR, we investigated the effect of IFN- on IDO and TDO expression in PHH and HepG2 cells. Unstimulated PHH and HepG2 cells expressed TDO but not IDO (Figure 6A). IFN-γ stimulation upregulated IDO expression in PHH and HepG2 cells (Figure 6B), but this did not increase Trp degradation via the Kyn pathway, further supporting our finding that Trp is degraded by TDO in PHH and HepG2 cells.

### 3.5. TDO Degrades L-Trp but Not D-Trp in HepG2 Cells

**Figure 7:**
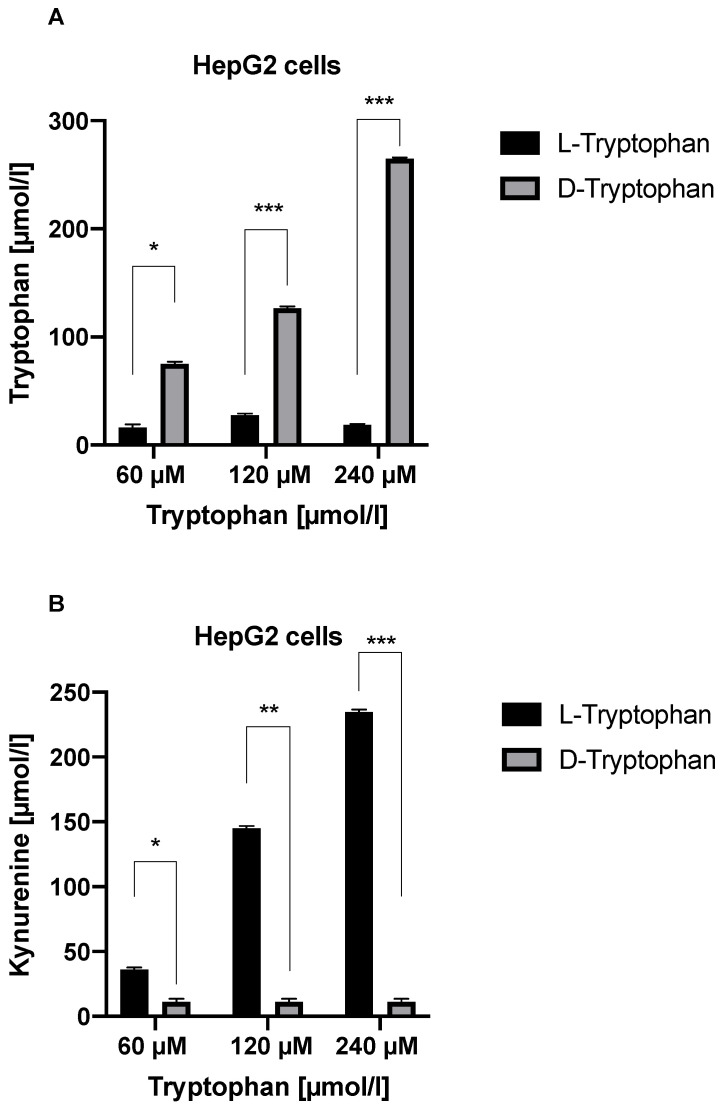
(**A**) Degradation of L-Trp and D-Trp by TDO in HepG2 cells. (**B**) Kyn accumulation from L-Trp and D-Trp degradation in HepG2 cells. * *p* < 0.05, ** *p* < 0.01, and *** *p* < 0.001.

IDO can degrade both the L- and D isoforms of Trp. To investigate which Trp isoform is degraded by TDO, we cultured HepG2 cells in media containing increasing concentrations of L-Trp or D-Trp. Interestingly, TDO was able to degrade L-Trp but not D-Trp in HepG2 cells (*p* < 0.05 for all concentrations) (Figure 7A,B).

## 4. Discussion

In this study, the involvement of TDO and IDO in Trp degradation and Kyn accumulation in PHH and HepG2 cells was investigated, with the aim of uncovering a potential therapeutic pathway for HCC immunotherapy. Our findings suggest that Trp is degraded to Kyn in liver tumor cells but that Kyn is metabolized further in normal hepatocytes. We also showed that the accumulation of Kyn in liver tumor cells is mediated by TDO, which degrades L-Trp but not D-Trp.

Currently, the multikinase inhibitor sorafenib is the only approved drug for treating advanced HCC (46), but some tumors develop resistance to sorafenib [22,23,24] because the degradation of Trp to Kyn suppresses T cells [25,26,27]. Thus, novel treatments for HCC that target and combat the immunosuppressive effects of Kyn accumulation are needed. Because Kyn increases immunosuppression by signaling through the aryl hydrocarbon receptor (AhR), targeting the IDO/TDO–AhR signaling pathway could lead to new anticancer therapies [28]. We used PHH and HepG2 cells as an in vitro model to investigate Trp metabolism in the present study. We showed that Trp was degraded in PHH and HepG2 cells, but that the rate of degradation was lower in HepG2 cells than in PHH cells. Interestingly, we observed an accumulation of Kyn in HepG2 cells, indicating that Kyn is the main metabolite of Trp degradation in liver tumor cells, even though the rate of Trp degradation is lower. The Kyn accumulation we observed in HepG2 cells suggests that liver tumor cells escape the host immune system by increasing Kyn levels. The enzymes TDO and IDO catalyze the degradation of Trp through the Kyn pathway [29] to induce effector T-cell apoptosis and immunosuppression [30,31]. Trp degradation and Kyn accumulation help tumor cells to evade the host immune system [32] by preventing the host immune cells from detecting and eradicating cancer cells [28]. We investigated whether IDO or TDO were responsible for these immune escape mechanisms in HCC cells.

The role of IDO in immunosuppression and tumor escape mechanisms has been confirmed in different cancers [28]. In contrast, we did not observe high IDO expression in normal hepatocytes or HepG2 cells, suggesting that IDO is not a suitable therapeutic target for HCC immunotherapy [33,34]. Indeed, our findings showed that IDO is not involved in Trp degradation in HCC cells. Even stimulating IDO expression with IFN-γ did not increase Trp degradation, which has also been observed in previous studies [35]. Although the role of Kyn and IDO in the pathogenesis and treatment of various cancers has previously been described [36,37,38], the role of TDO is less clear [39].

In this study, we show for the first time that two TDO inhibitors block Trp degradation and reduce Kyn accumulation in HCC-derived cells, and may represent candidates for HCC immunotherapy. These findings suggest that, unlike other cancer cells that respond to IDO inhibitors, HCC cells respond better to TDO inhibitors; moreover, these appear to be the most useful agents for treating HCC. A limitation of our study is that we did not assess the effect of Kyn accumulation on T-cell activity and immune responses in HepG2 cells. More research is needed to determine how inhibiting TDO affects tumor recurrence, patient survival, and tumor progression.

## 5. Conclusions

TDO plays a key role in Trp degradation in PHH and HepG2 cells. HepG2 cells produce high amounts of Kyn, which can lead to immunological resistance in HCC tissues. TDO inhibitors, such as 680C91 or LM10, may be useful in HCC immunotherapy because they prevent Kyn accumulation.

## Data Availability

Not applicable.
