# Correction: Oweira et al. Kynurenine Is the Main Metabolite of Tryptophan Degradation by Tryptophan 2,3-Dioxygenase in HepaRG Tumor Cells. *J. Clin. Med*. 2022, *11*, 4794

**DOI:** 10.3390/jcm12093177

**Published:** 2023-04-28

**Authors:** Hani Oweira, Imad Lahdou, Stefan Mehrle, Elias Khajeh, Rajan Nikbakhsh, Omid Ghamarnejad, Peter Terness, Christoph Reißfelder, Mahmoud Sadeghi, Ali Ramouz

**Affiliations:** 1 Department of Surgery, Medical Faculty Mannheim, University of Heidelberg, 68167 Mannheim, Germany; 2 Department of Transplantation Immunology, University of Heidelberg, 69120 Heidelberg, Germany; 3 Department of General, Visceral, and Transplantation Surgery, University of Heidelberg, 69120 Heidelberg, Germany

## Text Correction

There was an error in the original publication [1]. In all parts of the manuscript, the used cell line for the analyses was defined as “HepG2” instead of “HepaRG”. The corrections have been made to all sections of the manuscript by replacing “HepG2” with “HepaRG”.

The authors apologize for any inconvenience caused and state that the scientific conclusions are unaffected. This correction was approved by the Academic Editor. The original publication has also been updated.
